# Long-Duration Spaceflight Increases Depth Ambiguity of Reversible Perspective Figures

**DOI:** 10.1371/journal.pone.0132317

**Published:** 2015-07-06

**Authors:** Gilles Clément, Heather C. M. Allaway, Michael Demel, Adrianos Golemis, Alexandra N. Kindrat, Alexander N. Melinyshyn, Tahir Merali, Robert Thirsk

**Affiliations:** 1 Lyon Neuroscience Research Center, Bron, France; 2 Pennsylvania State University, University Park, Pennsylvania, United States of America; 3 International Space University, Strasbourg, France; 4 International Space University, Strasbourg, France; 5 McGill University, Montreal, Canada; 6 Western University, London, Ontario, Canada; 7 OrbitOne Consulting, Calgary, Canada; 8 University of Calgary, Calgary, Canada; Duke University, UNITED STATES

## Abstract

The objective of this study was to investigate depth perception in astronauts during and after spaceflight by studying their sensitivity to reversible perspective figures in which two-dimensional images could elicit two possible depth representations. Other ambiguous figures that did not give rise to a perception of illusory depth were used as controls. Six astronauts and 14 subjects were tested in the laboratory during three sessions for evaluating the variability of their responses in normal gravity. The six astronauts were then tested during four sessions while on board the International Space Station for 5–6 months. They were finally tested immediately after return to Earth and up to one week later. The reaction time decreased throughout the sessions, thus indicating a learning effect. However, the time to first percept reversal and the number of reversals were not different in orbit and after the flight compared to before the flight. On Earth, when watching depth-ambiguous perspective figures, all subjects reported seeing one three-dimensional interpretation more often than the other, i.e. a ratio of about 70–30%. In weightlessness this asymmetry gradually disappeared and after 3 months in orbit both interpretations were seen for the same duration. These results indicate that the perception of “illusory” depth is altered in astronauts during spaceflight. This increased depth ambiguity is attributed to the lack of the gravitational reference and the eye-ground elevation for interpreting perspective depth cues.

## Introduction

Depth perception is the visual ability to perceive the world in three dimensions and the distance of an object. In a recent investigation we found that the perception of distance and depth of objects was altered in astronauts after several months spent in weightlessness [1]. One interpretation for these changes is that the gravitational frame of reference on Earth influences the use of perspective and eye-ground elevation as depth cues. Indeed, the geometry of perspective projection is based on the presence of vanishing lines that intersect the projection plane at the horizon. Additionally, there is a tendency to perceive objects that are closer to the horizon as being farther away from us, and objects that are in our lower or higher part of the visual field as being closer to us [2]. The perception of depth from perspective and eye-ground elevation therefore requires a clearly identifiable horizontal and vertical coordinate system. In microgravity during spaceflight, the vertical direction of gravity can no longer be used as a reference. When astronauts are free-floating in the cabin, they generally report that the horizon is defined as being perpendicular to their longitudinal head or body axis, irrespective of their spatial orientation in the cabin [3]. When they anchor themselves to the ground using foot-straps, they tend to adopt a neutral body posture that is more flexed compared to normal gravity [4]. Therefore, the height between the eye and the ground can no longer be used as a reference for egocentric object distance.

**Fig 1:**
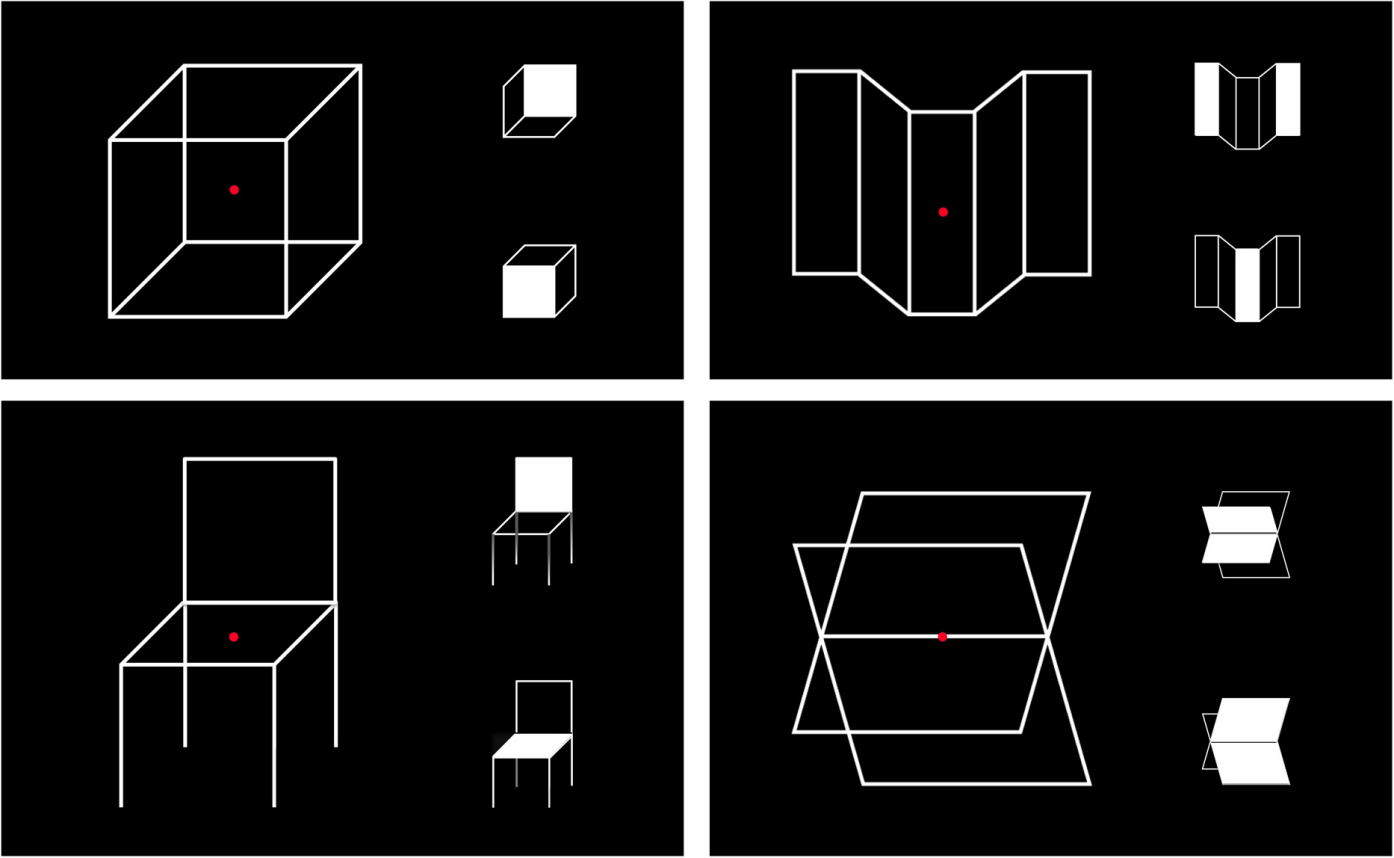
Reversible perspective figures used for testing depth ambiguity. Prior to the experiment, the subjects were informed about the two possible depth interpretations as shown in the inserts. The filled-in area(s) of the object appear(s) the closest to the observer. The inserts were not displayed during the actual tests. The percepts illustrated by the lower inserts were generally experienced first and the most often in normal gravity.

The objective of this study was to further investigate if the perception of depth was altered in astronauts during and immediately after spaceflight by using reversible perspective figures known to generate ambiguous illusions of depth. Perceiving depth in these figures is a form of illusion because the images are actually two-dimensional. The best known of these ambiguous figures is the Necker cube [5] that can lead to two different, mutually exclusive interpretations, i.e. a cube staying on a surface and seen from above, or a cube suspended from the ceiling and seen from underneath (Fig 1A).

Illusions that result from the difference between the objective and the subjective features of the environment are a common feature of visual perception. Consequently, illusions are a valuable tool for exploring the adaption of visual perception to unusual representation of the environments [6]. Based on the observations that perception of depth was altered in astronauts after long-term exposure to microgravity, we hypothesized a modified, and typically increased, depth ambiguity elicited by reversible perspective figures in astronauts in weightlessness.

Previous experiments have compared the perception of reversible figures in subjects upright and tilted supine or on their side, and concluded that the direction of the gravitational vertical influenced their responses [7, 8]. Experiments also have observed a decreased susceptibility to geometrical illusions in patients with signs of otolithic vertigo [9] or with lesions in the parieto-insular vestibular cortex [10, 11]. In the present experiment, we used ambiguous figures that could lead to two different, mutually exclusive depth interpretations. Depth ambiguity is maximal when each interpretation is perceived for half of the duration of the exposition. For reversible perspective figures such as the Necker cube, one interpretation is preferably seen in normal gravity, thus indicating less depth ambiguity [12]. By measuring the total duration that the reversible perspective figure appears to lie in each 3D orientation throughout the space mission, our objective was to establish whether depth ambiguity was reduced or increased during adaptation to weightlessness.

## Material and Methods

### Ethics Statement

This experiment was undertaken with the understanding and written consent of each subject. The test procedures were approved by and in compliance with the standards of the NASA Johnson Space Center Institutional Review Board for human testing and were performed in accordance with the ethical standards laid down in the 1964 Declaration of Helsinki.

### Subjects and Study Schedule

Six astronauts (5 males, 1 female) and 14 control subjects (8 males, 6 females), ranging in age from 22–53 years (mean 35.5 years) participated in this experiment. All subjects were tested during three sessions on the ground. For the control subjects, the mean interval between the first and the second session was 49 days, and the mean interval between the second and third session was 28 days. The astronauts were tested 10 times in total. They were first tested at approximately launch minus (L-) 220 days, L-160 days, and L-80 days. They were then tested on board the International Space Station during missions lasting 124–187 days (mean 152 days). The in-flight sessions took place on flight days (FD) 5–14 (mean FD08), FD20-36 (mean FD26), FD63-102 (mean FD72), and FD106-174 (mean FD134). Finally, they were tested again one day after return to Earth (R+1 day) and at R+5 and R+9 days (± 1 day depending on subject availability).

### Stimulus Figures

Two sets of figures were used in this study: the first set induced a depth ambiguity, such as a cube seen from above or from underneath; the second set generated an anthropomorphic ambiguity, such as a man’s head or a begging woman. Because the perception of these images temporarily changes back and forth even as one continues to look at the same stimulus figure, these images are referred to as reversible (or ambiguous) figures.

The figures that induced depth ambiguity were two-dimensional (2D) geometrical figures that elicited the perception of the same three-dimensional (3D) object in two different perspectives. These reversible perspective figures included the well-known Necker cube [5] and three other images found on the Internet (Fig 1). In these reversible perspective figures the 3D interpretation is derived solely from 2D shape cues. The figures were made of white lines on a black background: they did not have any gradient cues (shading, texture, occlusion) to convey 3D information, but instead relied on perspective. Note, however, that projection perspective was not actually used in these figures, as the edges of the receding sides of the 2D objects were drawn parallel, not converging. It is the *interpretation* of perspective, i.e. a mental construction of perceived depth, that can be reversed. Perceiving depth in these figures is a form of illusion because the figures themselves are actually flat.

Four other figures were used: the Mach book, the Ames trapezoidal window, the moving plaid [13], and the bi-stable see-saw [14]. However, these ambiguous figures elicited large intra- and inter individual differences in responses, with some subjects seeing only one interpretation while others saw more than two interpretations. Consequently the data corresponding to these latter figures were discarded from the analysis and are not reported here.

**Fig 2:**
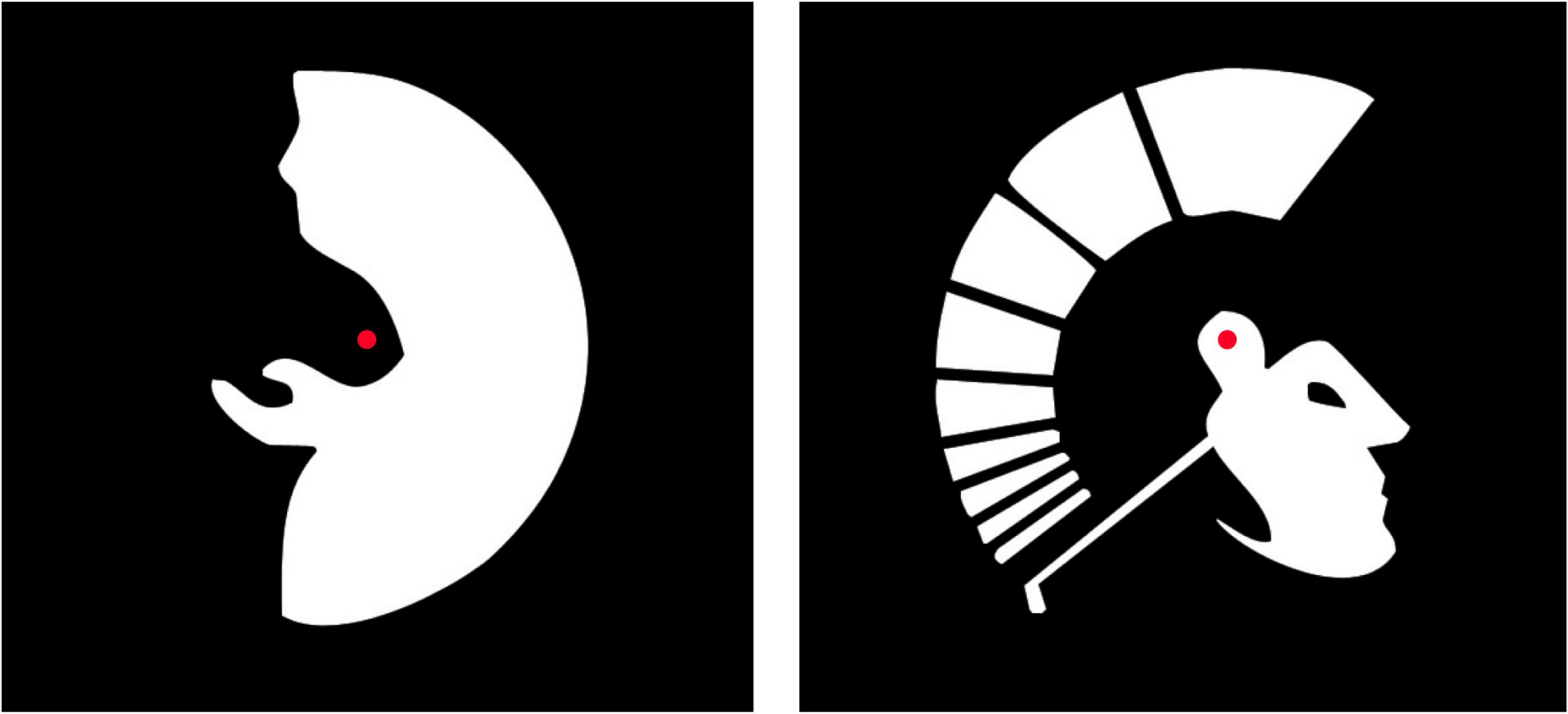
Silhouettes. These silhouettes can represent the profile of two different persons: a man’s face with a big nose or old woman begging with her hand extended (left); a Spartan soldier head and helmet or a golfer swinging (right).

The figures that induced anthropomorphic ambiguity were black or white silhouettes that elicited the reversibility of the meaningful content of what was seen: a man’s face or an old woman begging with her arm extended (based on [15]), a Spartan’s head with his helmet or a golfer swinging [16] (Fig 2). These figures are sometimes referred to as eliciting a “figure and ground effect” [17]. Note that only an anthopomorphic profile was seen in each of these figures, there was no depth information. These particular figures were also chosen because of the equal probability of spontaneous appearance of each of the two embodied anthropomorphic profiles upon first exposure [18].

Because it has been suggested that eye movements to different fixation points could cause reversals [19] a red dot was superimposed at the center of each figure. Subjects were instructed to fixate on this red dot to limit scanning eye movements.

### Experimental Protocol

**Fig 3:**
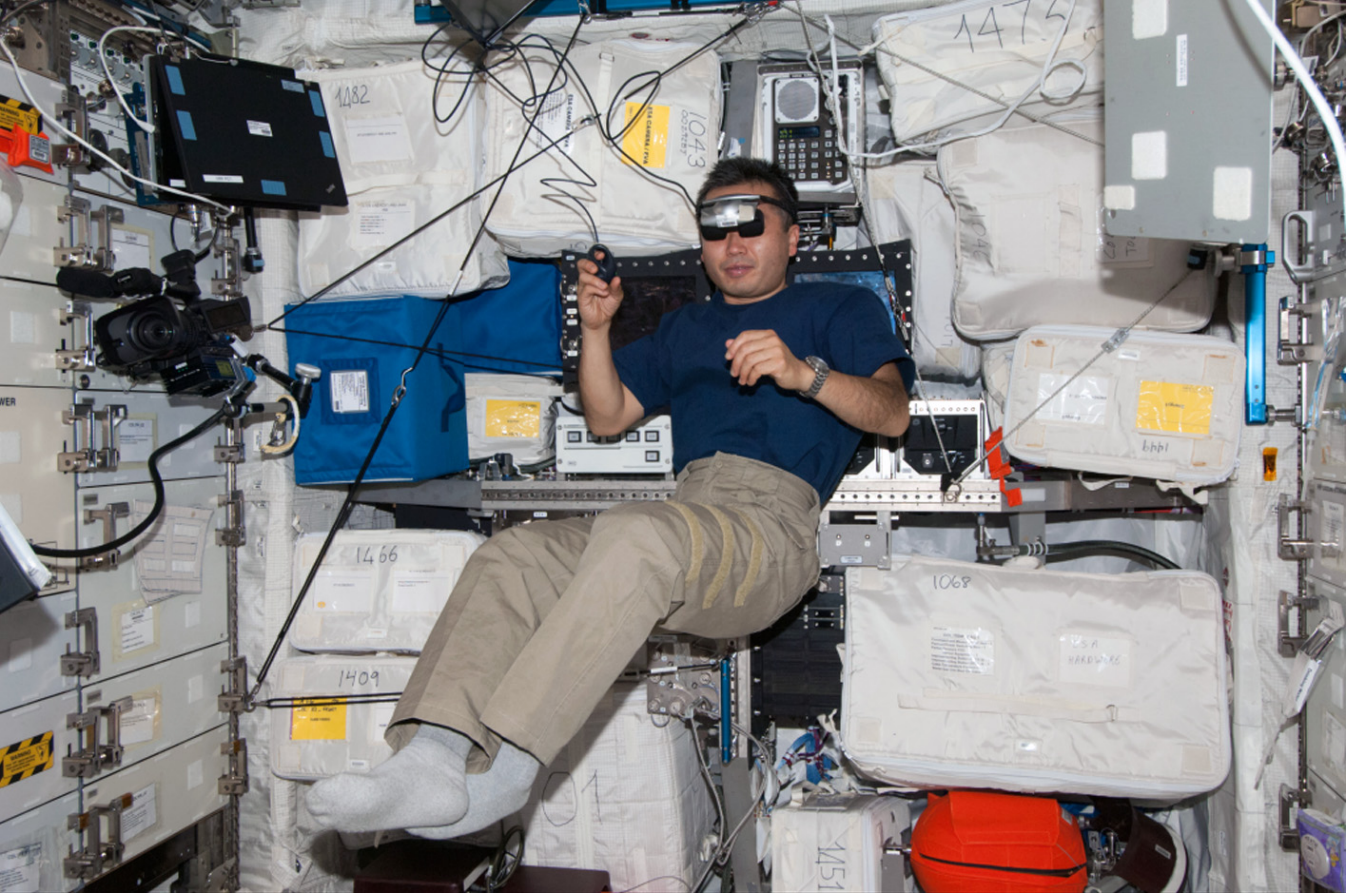
On board the International Space Station. An astronaut wearing the head-mounted display and holding a finger mouse in his hand is performing the experiment while free-floating. Photo credit NASA. The individual in this picture has given written informed consent (as outlined in PLOS consent form) to publish this photograph.

On the ground, subjects were tested when sitting at a desk. In orbit, the subjects were tested while free-floating to eliminate orientation-related cues (Fig 3). The figures were presented to the subjects in a head-mounted display (Z800 3DVisor, eMagin Corporation, Bellevue, WA) using a custom-made software running on a laptop computer. The figures subtended a viewing angle of 30° at a perceived distance of approximately 50 cm. Each figure was presented twice, for one minute each, in a random order. The refresh rate of the visual display was 60 Hz.

Subjects were informed about the two alternative perceptions beforehand. Since we were interested in studying perceptual reversals, we ensured that the subjects knew what the alternatives were. However, we also told the subjects that it was okay not to see the reversal, so that they did not assume that reversal was expected.

The subjects held a mini trackball finger mouse with two push buttons. The sample rate of the trackball was 40 Hz. To accurately record both the intervals of perceptual reversal and the perceived interpretation, subjects were asked to press the button on the finger mouse corresponding to the interpretation they perceived. Prior to each recording, subjects were shown the figure and a text message indicating the two different ways in which it could be seen (e.g., “chair with seat pointing towards you / away from you”; “man’s face with big nose / old woman begging”; etc.) and the corresponding clicking instructions (e.g. “right-click corresponds to the chair seat pointing towards you”; “right-click corresponds to the beggar”; etc.). Then the ambiguous figure was presented continuously for 1 minute. Subjects were asked to identify which interpretation they saw first, and then to repeatedly indicate when they saw the respective alternate interpretation by pressing down either of the two buttons of the finger mouse.

### Data Analysis

For each figure, the measurements included: (a) the reaction time, defined as the temporal period from stimulus onset to the subject’s first perceptual response; (b) the time to first reversal, defined as the temporal period from the subject’s first percept to the first reversal; (c) the number of reversals; and (d) the percentage of time for seeing each interpretation.

The measurements obtained in the two trials were averaged for each session and each subject. One-way ANOVAs were conducted to compare these measurements across the three sessions performed on the ground between the 14 control subjects and the six astronauts. This test was to verify that the responses of our small number of astronauts were within the range of a larger population. Within subjects (repeated measures) ANOVAs were then conducted to compare the effect of session days (10 sessions) and figures (2 silhouettes; 4 perspective figures) on the various response parameters. When a significant effect of days was seen, paired samples t-tests were used to make post hoc comparisons between the first session and the subsequent sessions.

## Results

### Reaction Time

**Fig 4:**
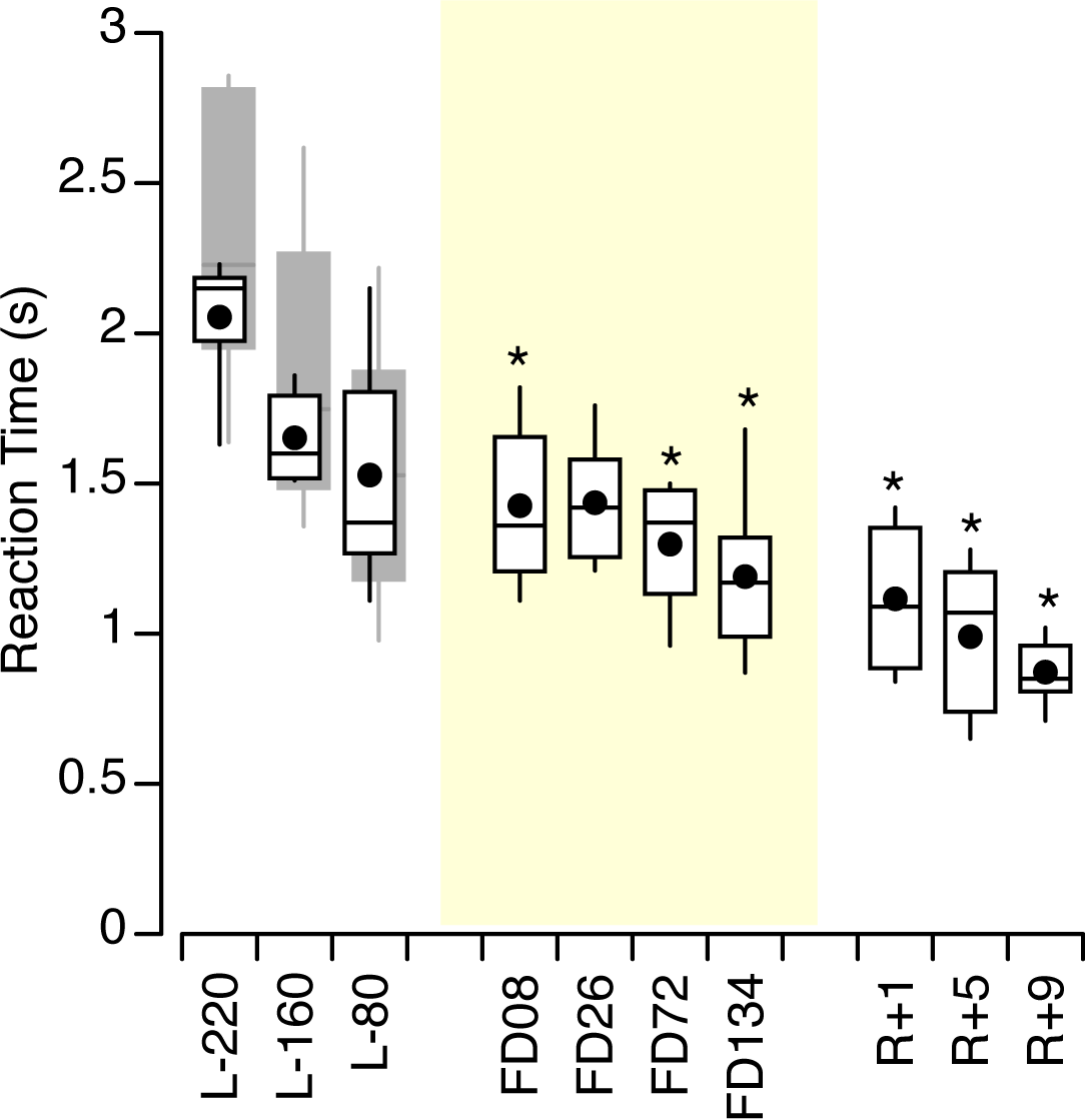
Reaction time. Duration between stimulus onset and the first response for all 6 figures for 6 astronauts across pre-flight (L-day), in-flight (FDday), and post-flight (R+day) sessions. Mean (black symbols) and median (horizontal lines) with 50^th^-percentile ranges (boxes) and 90^th^-percentile ranges (between whiskers); * *p* < 0.05 relative to L-220. The grey box-and-whisker plots show the responses for 14 control subjects.

During the tests performed on the ground, the reaction time was not significantly different between the control subjects and the astronauts [(F (1,359) = 3.38, *p* = 0.07]. A repeated-measures ANOVA in the control subjects’ data yielded a significant difference in reaction time across the 3 test sessions [F (2,234) = 5.92, *p* = 0.003] and the 6 figures [F (5,234) = 6.72, *p* < 0.001] but no interaction between the two [F (10,234) = 0.88, *p* = 0.82). A repeated-measures ANOVA in the astronauts’ data also yielded a significant difference in reaction time across the 10 test sessions [F (9,300) = 8.83, *p* < 0.001] and the 6 figures [F (5,300) = 9.50, *p* < 0.001] and no interaction between the two [F (45,300) = 1.20, *p* = 0.19). The reaction time decreased significantly between the first and the third session for the control subjects (paired t-test, *p* = 0.001) and with the repetition of the tests in-flight and post-flight for the astronauts (Fig 4).

### Time to First Reversal

**Fig 5:**
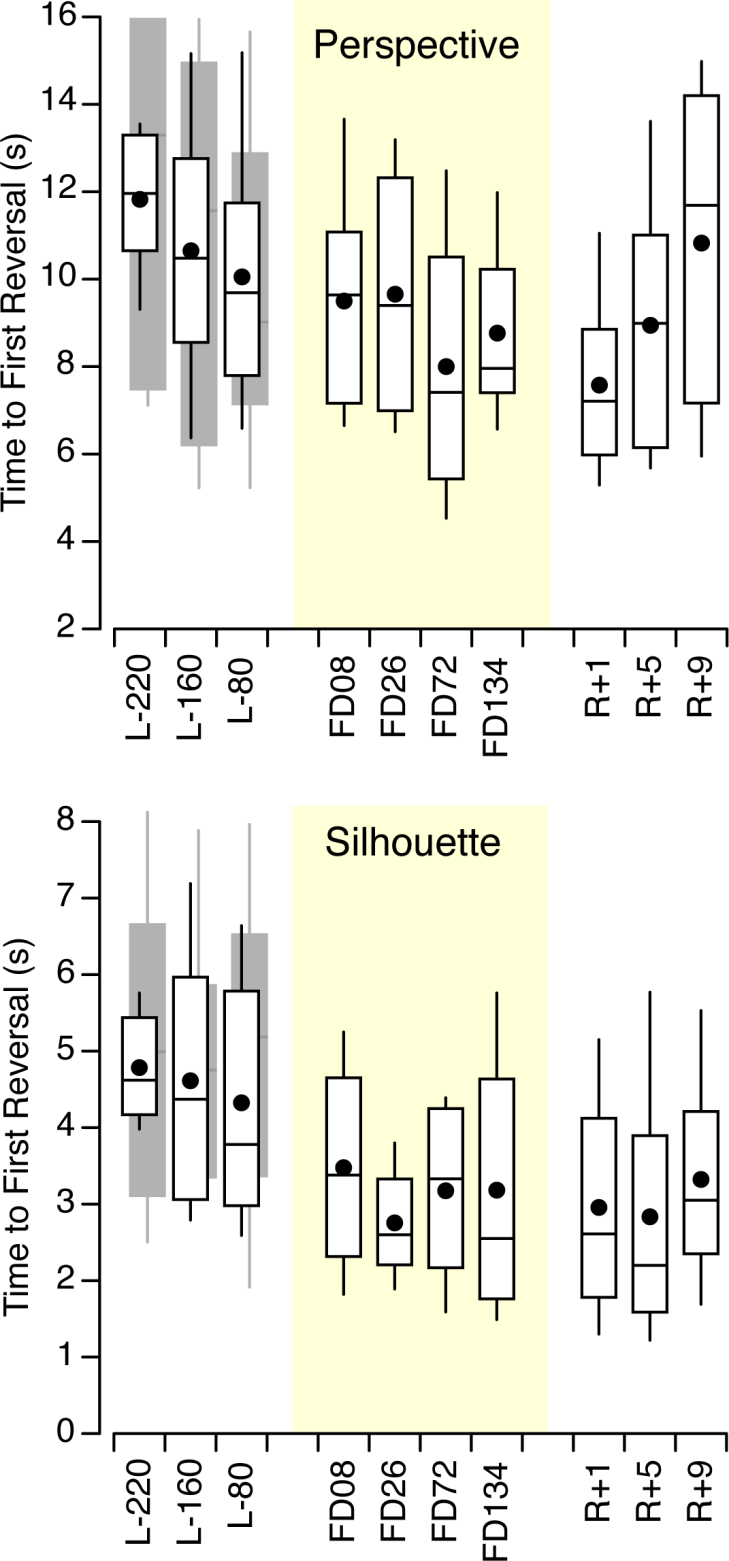
Time to first reversal. Time to first reversal for the four reversible perspective figures and the two reversible silhouettes for 6 astronauts (white box-and-whisker plots, mean and median) and 14 control subjects (grey box-and-whisker plots).

On the ground, the time to first reversal was not significantly different between the control subjects and the astronauts for both the silhouettes ([F (1,119) = 0.02, *p* = 0.33] and the perspective figures [F (1,239) = 0.73, *p* = 0.39]. However, the time to first reversal was longer for the perspective figures than for the silhouettes for both the control subjects [F (1,251) = 69.4, *p* < 0.001] and the astronauts [F (1,107) = 29.9, *p* < 0.001]. The time to first reversal averaged across all 20 subjects was 11.7 s (SD 9.1 s) for the perspectives figures and 5.1 s (SD 3.1 s) for the silhouettes. For both perspective figures and silhouettes, no significant difference was found across pre-flight sessions and figures for both the astronauts and the control subjects (Fig 5).

For the perspective figures, a two-way ANOVA in the astronauts’ data yielded no significant difference in the time to first reversal across the 10 sessions [F (9,239) = 0.83, *p* = 0.588] and the figures [F (3,239) = 2.42, *p* = 0.067]. No significant difference in the time to first reversal was observed for the silhouettes either, both across session days [F (9,119) = 1.08, *p* = 0.381] and figures [F (1,119) = 2.59, *p* = 0.111].

### Number of Reversals

**Fig 6:**
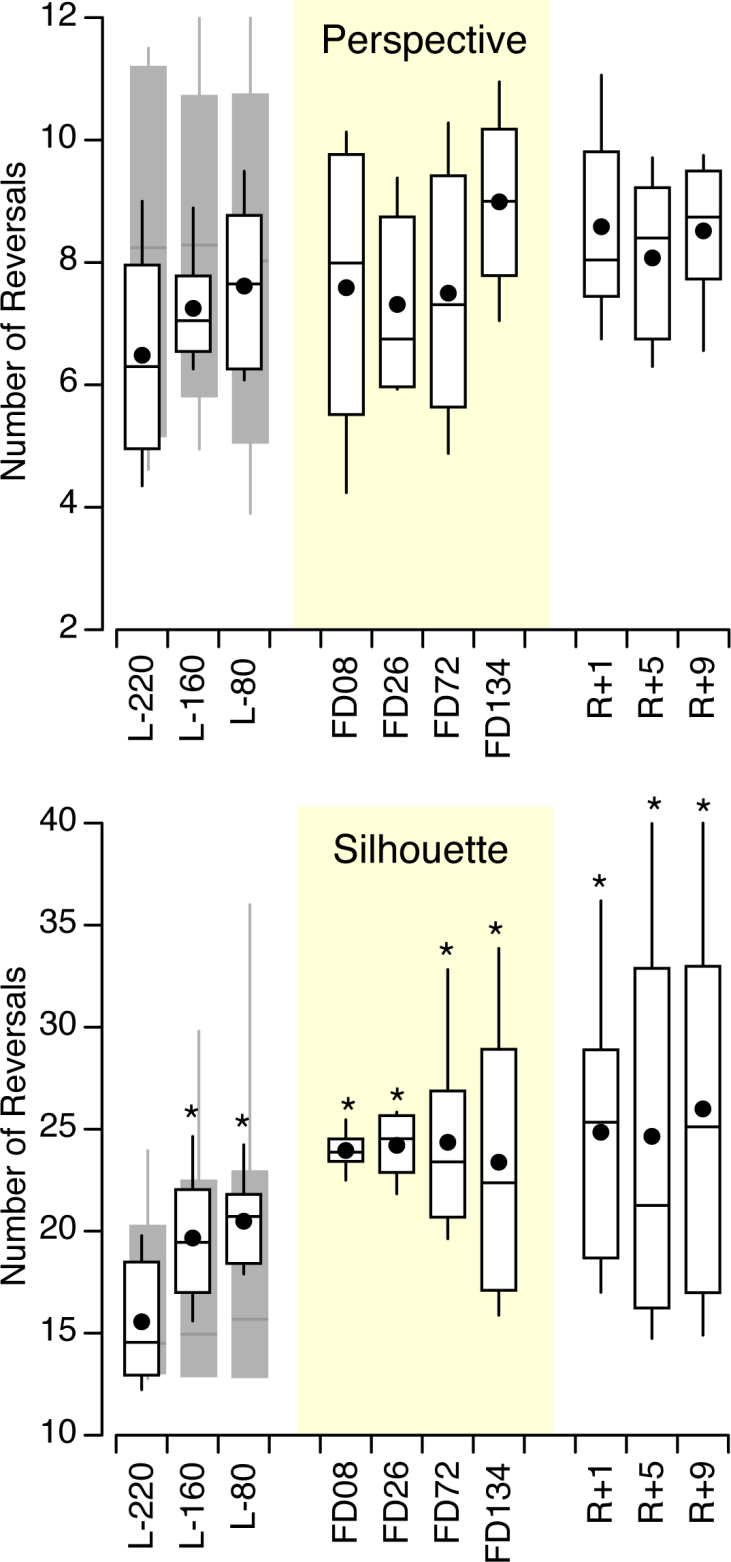
Number of reversals. Number of percept reversals per minute for the four reversible perspective figures and the two reversible silhouettes for 6 astronauts (white box-and-whisker plots, mean and median) and 14 control subjects (grey box-and-whisker plots); * *p* < 0.05 relative to L-220.

On the ground the number of reversals was not significantly different between the control subjects and the astronauts for the silhouettes ([F (1,119) = 0.66, *p* = 0.418], but was significantly different for the perspective figures [F (1,239) = 7.07, *p* = 0.008]. The number of reversals was smaller for the perspective figures than for the silhouettes for both the control subjects [F (1,251) = 59.6, *p* < 0.001] and the astronauts [F (1,107) = 213, *p* < 0.001]. The number of reversals per minute averaged across all 20 subjects was 8.7 s (SD 5.4 s) for the perspectives figures and 20.5 s (SD 15.4 s) for the silhouettes. For the perspective figures, no significant difference in the number of reversals was found across pre-flight sessions and figures for both the astronauts and the control subjects (Fig 6). However, for the silhouettes, the ANOVA indicated a significant effect of session days [(F (2,35) = 4.48, *p* = 0.019].

For the perspective figures, a two-way ANOVA in the astronauts’ data yielded no significant difference in the number of reversals across the 10 sessions [F (9,239) = 1.06, *p* = 0.392] and the figures [F (3,239) = 0.35, *p* = 0.788]. However, a significant difference in the number of reversals was observed for the silhouettes across session days [F (9,119) = 2.78, *p* = 0.006], but not across figures [F (1,119) = 0.08, *p* = 0.783]. The mean number of reversals increased from L-220 to FD08 and stabilized thereafter, but with a larger variability across subjects (Fig 6).

Because previous studies have reported an inverse correlation between the time to first reversal and the number of reversals [13, 20], we verified if this correlation was present in our data. The correlation coefficient between the time to first reversal and the number of reversals was 0.38 during the tests on the ground (mean of 20 subjects and 3 sessions) and 0.46 during the tests in-flight and post-flight (mean of 6 astronauts and 7 sessions).

### Percentage of Time for Seeing Each Interpretation

**Fig 7:**
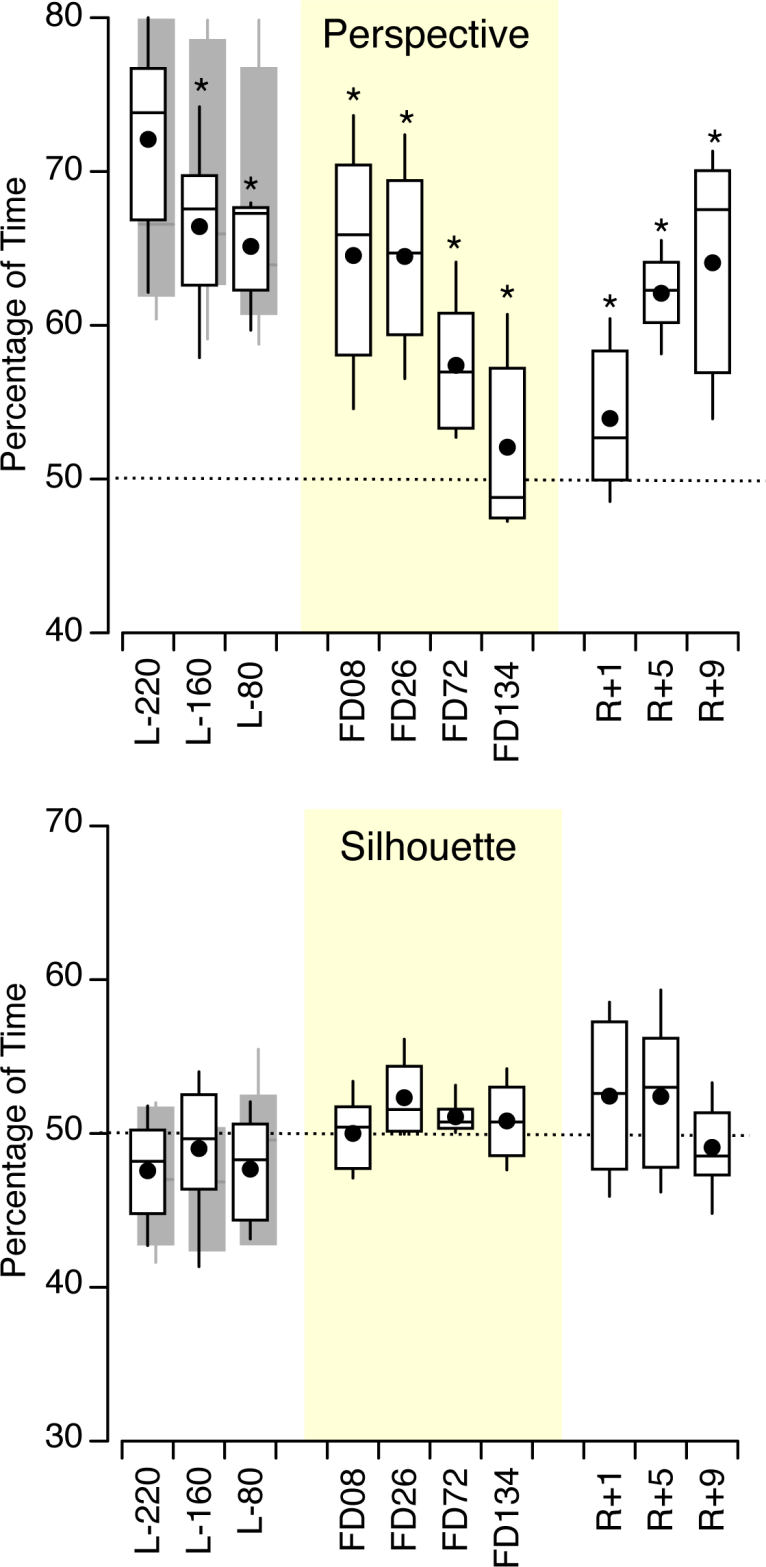
Percentage of time for seeing one particular interpretation. See text for details. The horizontal dotted line represents an equal duration for seeing each interpretation, i.e. perfect symmetry (50%); * *p* < 0.05 relative to L-220.

The percentage of time for seeing each interpretation was not significantly different between the control subjects and the astronauts for both the silhouettes [F (1,119) = 2.52, *p* = 0.115] and the perspective figures [F (1,239) = 0.855, *p* = 0.356]. However, there was a clear difference in the duration for seeing each interpretation between the silhouettes and the perspective figures for both the control subjects [F (1,251) = 66.8, *p* < 0.001] and the astronauts [F (1,107) = 65.2, *p* < 0.001]. When averaged across all 20 subjects the mean percentage of time for seeing the old woman and the golfer in the silhouettes was 50.5% (SD 11.9), i.e. close to chance. However, there was a 67%-33% asymmetry for seeing the perspective figures. The interpretations corresponding to the low inserts in Fig 1 were the most often seen. The mean percentage of time for seeing the Necker cube from above was 63.2% (SD 12.0%), the structure center popping out 65.8% (SD 12.3%), the large parallelograms in the foreground 66.8% (SD 14.3%), and the chair seat pointing toward the observer 71.2% (SD 14.3%). The interpretations that were seen for the longest duration were also the first interpretations that were seen on each trial (r^2^ = 0.74). Repeated measures ANOVA yielded no significant difference in the duration for seeing each interpretation across the 3 pre-flight sessions and the 6 figures for the control subjects, but a significant difference across the perspective figures for the astronauts [F (3,71) = 4.96, *p* = 0.004] (Fig 7).

For the perspective figures, a two-way ANOVA in the astronauts’ responses yielded a significant difference in the percentage of time for seeing each interpretation across the 10 sessions [F (9,239) = 4.55, *p* < 0.001] and the figures [F (3,239) = 10.09, *p* < 0.001], but no interaction [F (27, 239) = 0.68, *p* = 0.88]. The pre-flight asymmetry for seeing the perspective figures (67–33%) gradually decreased during the flight until reaching 52–48% on FD172, and re-increased after the flight (paired t-tests, *p* < 0.05) (Fig 7). No significant difference in the percentage of time for seeing each silhouette was observed across session days [F (9,119) = 1.22, *p* = 0.288] and figures [F (1,119) = 0.52, *p* = 0.471]. The first interpretations of the figures that were seen on each trial did not change significantly across the 10 sessions, for both the perspective figures [F (9,59) = 1.28, *p* = 0.269] and the silhouettes [F (9,59) = 1.04, *p* = 0.420].

## Discussion

Our results indicate that the reaction time progressively decreased with the repetition of the test sessions. The number of percept reversals of the anthropomorphic silhouettes progressively increased with the repetition of test sessions, but the number of reversals of the depth perspective figures was not altered by spaceflight. There was, however, a higher probability for seeing one of the two possible 3D interpretations in normal gravity. For example, when looking at the Necker cube on Earth, subjects saw a cube resting on a surface (i.e. as though viewed from above) more often that a cube hanging from the ceiling (i.e. as though viewed from below). Our results showed that this asymmetry progressively decreased in weightlessness as the flight progressed, and then returned to the preflight level after return to Earth.

Visual illusions are classically considered as a systematic mismatch between the basic response of the sensory organs to a stimulus (related to its physical properties), and the percept this object gives rise to. In the ambiguous figures, information is insufficient to result in a single interpretation, so these figures can be perceived as two different persons or two objects with different orientation [21].

The bi-stability of ambiguous figures is commonly thought to be the result of perceptual processes involving “bottom-up” messages (carrying sensory information) and “top-down messages” (carrying prior expectation). The percepts are the result of combining both types of messages [22]. The decrease in the reaction time confirms that an adaptation is taking place when subjects are repetitively exposed to ambiguous figures, and this adaptation is presumably dependent on sensory processes [23]. The continuous decrease in the reaction time also indicates that the changes in responses seen in our study were not due to subject fatigue or lack of concentration. As in previous investigations [13, 20], we observed that the time to first reversal and the number of reversals were two dependent measures. This is consistent with the interpretation of bottom-up processes underlying the adaptation effects [23]. Nevertheless, the observation that the mean duration of one given 3D percept was found to decrease over subsequent occasions while the reversal rate remained constant also indicates that the underlying percept reversal depends on two different processes. In addition, we observed that the first interpretation of the perspective figures when the stimulus was presented was also the interpretation most often reported during the trial. However, unlike the mean duration for seeing one 3D percept, the first interpretation did not change throughout the flight. This result supports the theory that “percept choice” at stimulus onset and “percept switching” after continuous observation are also two different processes [24].

The question asked in this experiment was whether the perception of *illusory* depth was affected in weightlessness. Weightlessness is characterized by changes in sensory information from gravity-sensing receptors (otolith organs, muscles and joint proprioceptors, and tactile pressure receptors), as well as alterations in the mental representation of three-dimensional space [3]. Actual changes in the perception of depth and distance of objects have previously been observed in astronauts during long-duration exposure to weightlessness [1]. The mean duration for seeing each 3D interpretation in the perspective figures progressively changed in the astronauts on board the ISS. This result indicates that the perception of illusory depth is altered in weightlessness. We argue that this adaption is due to the lack of both a gravitational reference and the eye-ground elevation reference, which reduce the salience of depth cues.

### Vertical and Depth are Related to Gravity

Under terrestrial conditions, depth corresponds to the distance relative to the observer, in the direction of the line of sight; width, i.e. the line joining the two eyes, corresponds to the horizon; and height corresponds to the direction of gravity. In a drawing, vertical lines represent either the direction of the terrestrial gravitational vertical or the direction of depth: the farther away an object is, the higher it is represented on the page. Thus, there is therefore confusion between up-down and fore-aft. Such confusion is reflected in illusions of distortions, such as geometrical illusions, and in illusions of ambiguities, such as figure/ground illusions and ambiguous figures.

Geometrical (distortion) illusion figures are essentially skeleton perspective drawings suggesting depth. When the illusion is about judgment of parallelism or alignment, it is minimal for horizontal and vertical orientations and maximal for oblique orientations (±45°). In judgments of size, we are also less accurate for the oblique directions. For example, the oblique lines in the Muller-Lyer and the Ponzo illusions and the vertical line in the horizontal-vertical illusion all give the same projection as the receding lines, for example, of a railway [21]. These illusions also depend on the orientation of the head relative to gravity [7, 8]. The problem seems to be in the tendency for the visual system to refer everything to the direction of the gravitational vertical [25].

Gibson [26] interprets the apparent distortion in geometrical illusions in the context of size constancy, and suggests that perception of size is a by-product of a constant scale, which Gregory [27] calls “constancy scaling”, at different distances. Constancy scaling is the tendency to perceive an object with its intrinsic qualities (properties) rather than its changing aspects. Arguing that illusory figures are “flat projections of typical views of objects lying in three-dimensional space”, Gregory notes that “the parts of the figure corresponding to distant objects are expanded and the parts corresponding to nearer objects reduced”. Gregory therefore postulates a common process modifying retinal images in constancy scaling and in the interpretation of illusory figures.

### Perception of Depth in Astronauts and in Patients

The “visual cliff” studies have shown that the capacity to perceive depth is present from the beginning of an organism’s life [28]. However, other investigations indicate that learning plays a role in the development of depth perception. After a short period of exposure to conflicting cues, such as wearing prisms that tilt the images, it has been shown that observers recalibrated the depth implied by the vertical lines. An opposite effect was seen after the prisms were removed [29].

Anthropological studies have also shown that geometrical illusions were more prevalent in people living in the constructed environment of cities than those dwelling in more natural environments [30]. Although we perceive the location of objects in the third dimension based on certain innately given cues, we also learn to use additional cues and learn to interpret given cues with greater precision after birth.

In a normal gravity environment, we are more likely to see a cube resting on a table than hanging from the ceiling; hence the longer duration for seeing a cube from above when looking at the Necker cube. Another well-known example of knowledge built into our visual system is the assumption that scenes are illuminated from above [31]. An elegant experiment performed on board the Neurolab mission in 1998 indicated that this shading illusion is absent in astronauts in weightlessness, presumably because they get used to receiving light from any directions when they are free-floating in the spacecraft cabin [32]. This indicates that a relatively high level of processing is at the source of the perceptual bias.

Geometrical illusions were found to generate less distortion in astronauts during spaceflight [33]. The number of reversals of ambiguous figures was found to decrease in parabolic flight [34] and to increase in orbital flight [20]. The conditions of these earlier tests were quite different from those in the current study, which could explain these discrepancies. For example, the subjects had received less training and no fixation points were used on the figures. The transitions between high and low gravitational loads in parabolic flight are known to generate vertical gaze deviations that could have confounded the results [20].

Ground-based experiments have also shown a decreased susceptibility to geometrical illusions in vestibular patients [9]. Patients who suffer from otolithic vertigo are significantly less sensitive to illusions based on size and depth. When presented with reversible perspective figures (e.g. the Schroeder staircase or the Necker cube) these patients are less biased by the perspective effect and thus report only one of the possible percepts. Patients with lesions in the parieto-insular vestibular cortex are also less sensitive to geometrical illusions [10, 11]. Other investigations have shown that three to four-year old children reversed ambiguous figures only once or twice over a 60 s presentation period, even when informed about the two possible interpretations [35]. However, children between the ages of 7 and 9 reversed ambiguous figures as adult participants did [36]. Willats [37] argues that the ability to use projection systems (i.e. to draw diagonals as if objects were seen from above) in drawings of 3D objects is achieved with an onset between 9 and 10 years of age. These studies point out that perceived depth through perspective is partly learned by our experience of living in a gravitational environment.

It was also observed that blind people who recovered sight later in life [17] and patients with schizophrenia [6] were not sensitive to ambiguous figures or depth-based geometrical illusions. These investigators empirically interpreted this phenomenon as a sign of a reduced top-down influence in visual perception. Use of magnetic resonance spectroscopy has revealed that patients who suffer from schizophrenia exhibited a reduced GABA concentration in the visual cortex compared with healthy controls [38]. GABA has also been shown to contribute to visual perception processing of reversible figures [39]. The investigators observed a lengthening of the percept durations and a decrease in the reversal rates after the systemic administration of a GABA agonist (lorazepam). Because GABA transmission reflects inhibitory neuronal modulations, these findings are another argument in favor of a reduced influence of top-down processes in subjects who are less sensitive to reversible perspective figures. Also, recent experiments have shown a relationship between GABA levels and perceived duration of time intervals [40]. Alteration in time perception has been casually reported by cosmonauts during spaceflight [41], which might impact the perception of temporally interleaved ambiguous patterns [42]. The authors of this paper have an on-going ISS experiment investigating whether the subjective perception of time is actually affected by weightlessness. The outcomes of this experiment should clarify the relationship.

### Eye-Ground Elevation as a Depth Cue

Another pictorial source of depth information that arises from perspective projection is the height of objects in the picture-plane relative to the horizon. Because humans are bipedal creatures standing upright in normal gravity, the objects located in the lower part of our visual field (i.e. at our feet) are perceived to be closer to us than those in the higher part of our visual field (i.e. at the ceiling or in the sky) [2]. It can therefore be argued that we preferentially see the Necker cube from above because the face of the cube that is the closest to us is located in the lower part of our visual field (see the lower insert for the Necker cube in Fig 1). This perception is also related to our visual experience of seeing objects below our line of sight while standing in Earth’s gravity [43]. Our visual estimates of distance, height and size of objects have been compared countless times since early childhood with visual experience and the kinesthetic information resulting from bodily contacts with the objects [44].

After adaptation to weightlessness, free-floating astronauts have become accustomed to seeing the visual environment from various viewpoints. The objects in the lower part of their visual field are not necessarily the closest. Also, their feet are not always in contact with the “floor” and they do not maintain an upright posture [4]. Consequently, the eye-ground elevation (height) can no longer be used as a reference for estimating distance from eye level. Also, the inter-aural axis is not necessarily aligned with the horizon, and changes in the perceived straight-ahead might occur [3]. We therefore argue that some cues to depth depend on gravity. On Earth, the gravitational vertical reference and the eye-ground elevation presumably contribute to bias the ambiguity of depth in reversible perspective figures. In weightlessness, this biased ambiguity progressively disappears and after three months in space, the time taken for each reversal is the same, as is the case for ambiguous figures with no depth cues.

## Conclusion

Theorists like Gibson have argued that the core of our perception of the third dimension is our interpretation of planar surfaces such as the ground rather than our interpretation of objects separated from one another in empty space. Of all the cues we know about, only perspective could provide direct information about planes and their orientation with respect to us. However, the contribution of perspective is not a necessary factor, since the objects look three-dimensional even when linear perspective is eliminated (in the reversible perspective figures used for this study, the edges of the receding sides were drawn parallel, not converging).

During the vast majority of everyday life situations on Earth, we resolve the ambiguity of the environmental stimuli through sensory information and prior knowledge. After adaptation to weightlessness, astronauts develop larger depth perception instability, manifested by an equal probability for seeing each 3D interpretation after three months in space. This perceptual instability is presumably due to impairment in sensory information related to the spatial orientation relative to the vertical and in top-down processes implicitly driven by prior normal gravity knowledge. This model may also account for the cognitive deficits observed in what the astronauts call the “space fog” syndrome, which is characterized by difficulties in correctly allocating attention and filtering out irrelevant information [3].

These observations confirm that depth is not so much the direct result of certain specifiable stimulus cues as it is a mental construction. This mental construction would obey some rules related to our experience of living in a gravitational environment. The vertical reference, the eye-ground elevation, and the size-constancy scaling are presumably some of the rules that the visual system utilizes to interpret perspective cues to indicate distance and depth.
